# Decreased chlamydia notifications in six European Union/European Economic Area countries and England, 2024

**DOI:** 10.2807/1560-7917.ES.2026.31.21.2500846

**Published:** 2026-05-28

**Authors:** Ana Martina Astorga Alsina, Joana Gomes Dias, Cornelia Adlhoch, Lina Nerlander, Anne Olaug Olsen, Derval Igoe, Eija Hiltunen-Back, Fiona Lyons, Inga Velicko, Kate O'Donnell, Kate Folkard, Katerina Crawford, Kirsi Liitsola, Maartje Visser, Maria Wessman, Martha Neary, Miranda Ferguson, Mirja Puolakkainen, Steen Hoffmann, Thomas Roland Pedersen, Tuula Hannila-Handelberg, Otilia Mårdh

**Affiliations:** 1 European Centre for Disease Prevention and Control (ECDC), Stockholm, Sweden; 2 Norwegian Institute of Public Health, Oslo, Norway; 3 HSE Public Health: National Health Protection Office, Dublin, Ireland; 4 Finnish Institute for Health and Welfare, Helsinki, Finland; 5 HSE Sexual Health Programme, Dublin, Ireland; 6 Public Health Agency of Sweden, Stockholm, Sweden; 7 HSE Health Protection Surveillance Centre, Ireland; 8 UK Health Security Agency, London, England; 9 National Institute for Public Health and the Environment, Bilthoven, the Netherlands; 10 Statens Serum Institut, Copenhagen, Denmark; 11 University of Helsinki, Helsinki, Finland

**Keywords:** *Chlamydia trachomatis*, surveillance, epidemiology, Europe

## Abstract

Over the last decade, bacterial sexually transmitted infection notifications have increased across the European Union/European Economic Area (EU/EEA) and England. However, six EU/EEA countries — Denmark, Finland, Ireland, the Netherlands, Norway and Sweden — and England, reported declines in chlamydia notifications from 2023 to 2024. To explore potential drivers of these decreases, the European Centre for Disease Prevention and Control, together with experts from the seven countries, analysed national surveillance data from 2015 to 2024 and contextualised findings with information on testing practices, policies and public health interventions. Chlamydia notifications in 2024 were compared with expected counts based on trends before the COVID-19 pandemic (2015–2019) using a negative binomial model. Across countries, decreases between 2023 and 2024 ranged from 13% to 19%, primarily affecting 15–24-year-olds. Case numbers fell below the 95% prediction interval in three countries. Five countries reported reduced testing volumes among young people, and all observed lower positivity rates. Some countries implemented online testing and targeted sexual health promotion campaigns. Although reduced testing, changes in behaviour during the COVID-19 pandemic and the risk profile of those accessing testing may have contributed to the declines, further investigation into underlying drivers is necessary to inform sexual health strategies in Europe.

## Background

*Chlamydia trachomatis* infection is the most frequently reported sexually transmitted infection (STI) in the European Union/European Economic Area (EU/EEA), with 230,119 notifications in 2023 from 27 Member States [1]. Young people (15–24-years-old) accounted for 56% of cases with known age (122,500/218,809). Among cases with reported transmission category, heterosexual transmission predominated (79%; 58,400/73,888), followed by transmission among men who have sex with men (MSM) (20%; 14,800/73,888) [2]. Across the EU/EEA, country-specific notification rates differ substantially, ranging from 0.1 cases per 100,000 population in Romania to 625.5 in Denmark in 2023, reflecting differences in testing practices, control policies and surveillance systems [3]. Between 2014 and 2023, notification rates generally increased in most of the 20 countries in the EU/EEA with consistent reporting and comprehensive surveillance, seeing a first peak in 2019, a decline during the COVID-19 pandemic years (2020 and 2021), a rebound in 2022, and slight decrease in 2023. In England, chlamydia epidemiology showed a similar trend between 2015 and 2021 but remained stable after a 2022 rebound [4].

In early 2025, Sweden reported a nation-wide decrease in notified chlamydia cases in 2024 compared with 2023 through EpiPulse Events (a European Union event-based surveillance platform hosted by the European Centre for Disease Control and Prevention (ECDC)) [5]. In EpiPulse Events, representatives of public health authorities in the EU/EEA and other countries, including the United Kingdom (UK), may initiate alerts and/or comment on ongoing events. Sweden indicated that the decrease was mainly among young people despite unchanged testing policies. Denmark, England, Finland, Ireland, Norway and the Netherlands replied with comments, reporting similar trends. Chlamydia surveillance is comprehensive and laboratory-based in Denmark, England, Finland, Ireland, Norway and Sweden, using molecular testing (i.e. nucleic acid amplification tests (NAATs)) for case confirmation. The Netherlands employs a sentinel surveillance model that collects data on diagnosis counts and case characteristics from all national sexual health centres (SHCs) serving at-risk populations.

To better understand the recent decreases and explore their potential drivers, ECDC facilitated an exchange of epidemiological data and working hypotheses among these seven countries. In this report, each of the seven countries present an analysis of chlamydia notification data and trends between 2015 and 2024. In addition, we summarise hypotheses proposed and investigated by national experts on potential drivers behind the observed declines. The findings may contribute to the body of evidence required to inform and tailor national prevention strategies.

## Chlamydia trends 2023–2024

In this section, the seven countries describe chlamydia trends based on national surveillance data and contextualise those with country-level information on testing and public health interventions.

### Common methodology

The countries used a common methodological approach, involving the analysis of national surveillance data on *C. trachomatis* infections from 1 January 2015 to 31 December 2024 to describe changes in case notifications by year, sex (male or female) and age group (0–14, 15–19, 20–24, 25–34, 35–44 and ≥ 45 years). In addition, information on epidemiological trends, case characteristics and testing rates was extracted from national reports where available [6-12]. National experts shared contextual information on recent changes in public health interventions and working hypotheses through the EpiPulse Events platform. These were further discussed during a joint teleconference organised by the ECDC in March 2025, with participation from Denmark, Finland, Ireland, the Netherlands, Norway and Sweden. England contributed input at a later date.

Methodological aspects specific to an individual country are described in the respective country chapters.

### Chlamydia trends by country

**Figure 1:**
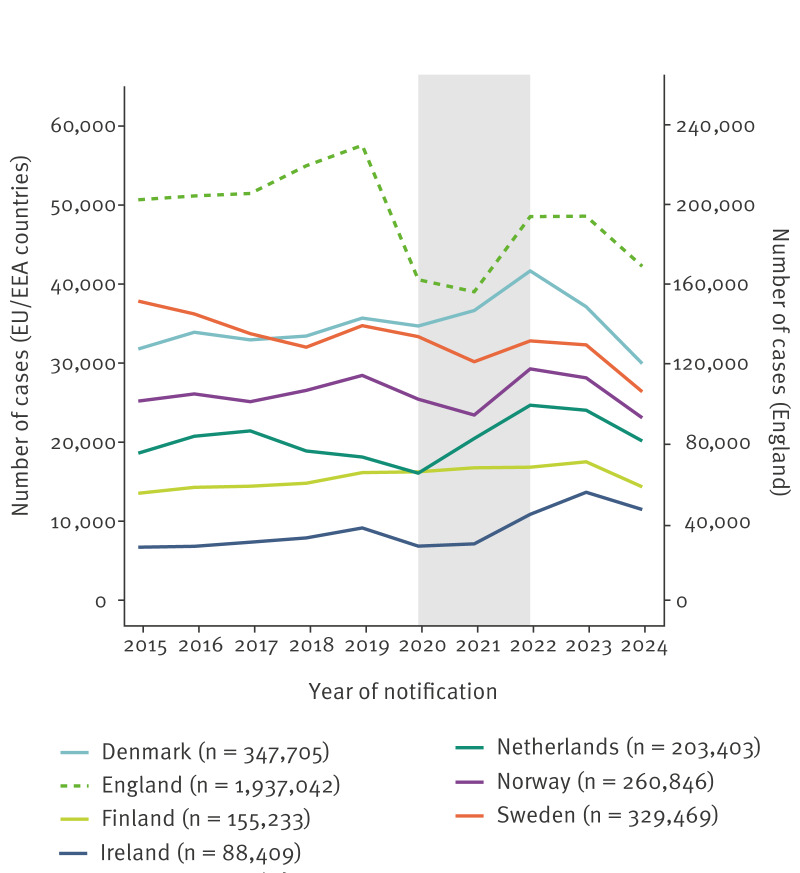
Chlamydia case notifications by country and year, 2015–2024 (n = 3,322,107)

**Figure 2:**
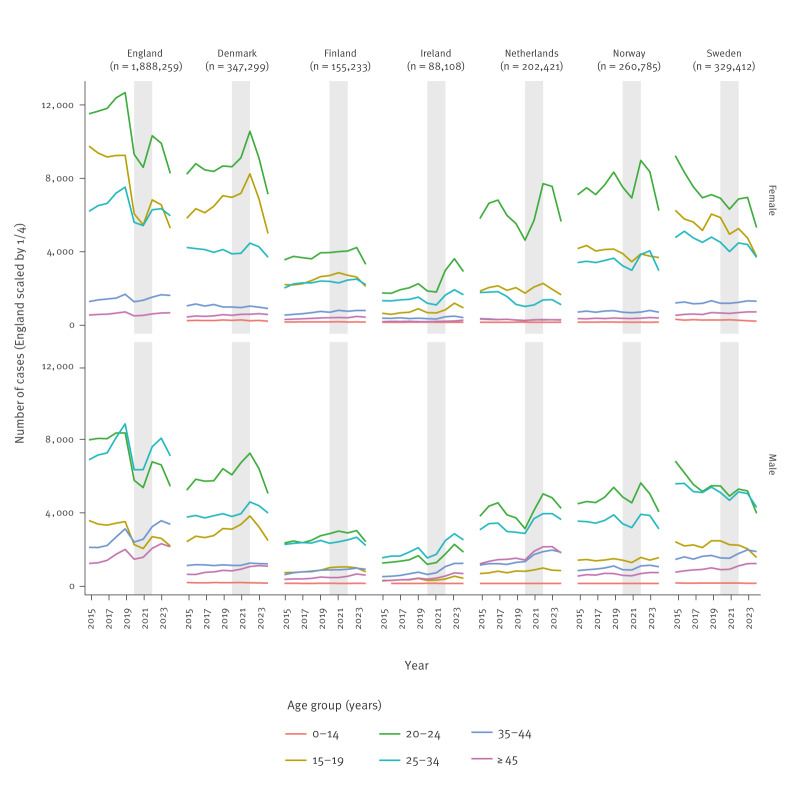
Number of chlamydia case notifications per country by sex, age group and year, 2015–2024 (n = 3,271,517)

In 2024, all seven countries observed an overall decrease in chlamydia cases compared with 2023 for both males and females aged 15–45 years, with reductions ranging from 13% to 19%. The largest declines were observed among those aged 15–24 years (Table 1; Figures 1–2). In earlier years, case numbers showed low-level year-to-year fluctuations, declined in most countries during the COVID-19 pandemic (2020 and 2021), and then increased again in 2022 and 2023.

**Table 1:** Surveillance trends of notified chlamydia cases by country, geographical extent of chlamydia trends, trends by population subgroup, and reported changes in testing and public health activities, 2023–2024 (n = 641,387)

Country	Denmark	Finland	Ireland	The Netherlands	Norway	Sweden	England
STI surveillance	Co, Cp, laboratory	Co, Cp, laboratory	Co, Cp, laboratory and physicians	Sentinel	Co, Cp, laboratory	Co, Cp, laboratory and physicians	Co, Cp, laboratory and physicians
Surveillance trends 2023 to 2024. Source: national surveillance data, EpiPulse Events, national reports							
Chlamydia	Decrease of 19%, 37,111 to 29,946 cases	Decrease of 17%: 17,551 to 14,486 cases	Decrease of 16%: 13,699 to 11,524 cases	Decrease of 16%: 24,048 to 20,174 cases	Decrease of 18%: 28,137 to 23,100 cases	Decrease of 18%: 32,298 to 26,392 cases	Decrease of 13%: 194,143 to 168,889 cases
Geographical extent of chlamydia decrease	All regions; decrease range: 12–32% (sex-specific)	21 of 23 regions; decrease range: 9–34%	All regions	All regions	All counties	19 of 21 regions; decrease range: 10–42%	All regions
Population groups affected by chlamydia. Source: national surveillance data, EpiPulse Events, national reports							
Age group	Decrease driven by those aged 15–24 years	Largest decrease among those aged 20–24 years	Largest decrease of 21% in those aged 15–24 years	NA	Largest decrease of 22% in females aged 20–24 years and 19% in males aged 20–24 years	Largest decrease in those aged 15–24 years	Largest decrease in 15–24 years age group (18%)
Sex	Decrease of 22% (F), 17% (M)	Decrease of 21% (F), 20% (M)	Decrease of 19% (F), 13% (M)	Decrease of 23% (F), 13% (HTXM)	Decrease of 20% (F), 14% (M)	Decrease of 20% (F), 17% (M)	Decrease of 14% (F), 13% (M)
MSM	NA	NA	NA	Decrease of 15% (MSM-PrEP), 6% (MSM-regular)	NA	Proportion of cases stable at 17% of male cases	Decrease of 11% in gbMSM
Chlamydia testing (2023 and 2024). Source: EpiPulse Events, national reports							
Testing activity	Decrease (6%): 295,346 to 277,862 tests	Slight decrease (2%): 359,958 to 352,908 tests	NA	Decrease (9%): 172,113 to 157,386 tests; subgroup-specific decreases of 3–25%	Decrease (5%): 390,948 to 377,422 tests; decrease in < 25-year-olds	Slight increase (1%): 593,887 to 598,118 tests	Slight decrease (1%): 3,266,474 to 3,240,203 tests; decrease (11%) in 15–24-year-olds: 978,148 to 874,194 tests
Positivity rate	Decrease in both sexes, with largest reductions in ages 15–19 and 20–24: men (27.9% to 25.8%; 21.6% to 19.4%), women (18.0% to 15.8%; 11.4% to 10.4%)	Decrease observed in six major laboratories; no additional data available	Decrease from 7.1% to 6.1% (home testing); no data available for other test settings	Decreases across all groups: women (16.8% to 15.3%), HTXM (19.6% to 19.2%), MSM-PrEP (9.1% to 8.3%), MSM-regular (10.2% to 9.3%)	Decrease mainly in ages 15–19 and 20–24: men (15.2% to 14.2%; 14.9% to 13.3%), women (10.9% to 9.5%; 9.0% to 7.6%)	Decrease overall from 5.4% to 4.4%	Decrease overall from 5.9% to 5.2%. Among individuals aged 15–24 years, decrease from 10.6% to 9.8%
Testing policy change	No change	No change	No change	Pharyngeal testing discontinued for MSM/women; asymptomatic testing restricted in pilot regions	No change	No change	In 2021, screening policy changed from offering asymptomatic testing to all those aged 15–24 years to only women aged 15–24 years
Public health activities. Source: EpiPulse Events, national reports							
Notable changes in public health activity since 2022	Targeted public health campaigns for young people	No major changes reported in recent years	Home testing (≥ 17 years) introduced in 2022, and expanded, targeted media campaigns during 2023–2024	No major changes reported in recent years	Targeted public health campaigns on testing, condom use and sexual health awareness, especially among students	No major changes reported in recent years	No major changes at national level, however, local areas will have undertaken health promotion activity in response to STI outbreaks/increases

Co: comprehensive; Cp: compulsory reporting; F: female; M: male; HTXM: heterosexual men; Laboratory: laboratory reporting; MSM: men who have sex with men; gbMSM: gay, bisexual and other men who have sex with men; MSM-PrEP: MSM using pre-exposure prophylaxis for HIV; MSM-regular: MSM attending for regular care; NA: data not analysed or not available; NA: not applicable; Physicians: data collected from physicians; Sentinel: sentinel surveillance system; STI: sexually transmitted infection.

EU/EEA: European Union/European Economic Area.

Grey area: COVID-19 pandemic years 2020–2022.

Grey area: COVID-19 pandemic years 2020–2022; y-axis (England data) scaled 1/4; England did not provide data for the 0–14 age group because of information governance restrictions.

#### Denmark

Chlamydia cases declined by 19% between 2023 and 2024 (from 37,111 to 29,946 cases) (Table 1), with 17% and 22% declines in males and females, respectively. Case notifications declined between 12% and 32% across all regions [11,13]. The decrease mostly affected young people aged 15–19 years (−27%) (Figure 2). In 2024, testing activity decreased by 6% compared with 2023 and positivity rates declined among young people in both males and females (Table 1).

No testing policy changes were implemented in Denmark, but several sexual health campaigns were launched following the rise in gonorrhoea cases in 2022, particularly targeting younger age groups.

#### England

For England, data were derived from the Sexually Transmitted Infections and National Chlamydia Screening Programme (NCSP) Annual Data and related reports [4,14-16]. These data tables and reports contain aggregate data from all publicly funded chlamydia and gonorrhoea tests and diagnoses reported to the Genitourinary Medicine Clinic Activity Dataset (GUMCAD) STI Surveillance System or the Chlamydia Testing Activity Dataset (CTAD) Chlamydia Surveillance System.

Chlamydia cases decreased by 13% in 2024 compared with 2023 (194,143 to 168,889 cases; Table 1), across sexes, age groups and regions in England. The largest decrease (18%) occurred in individuals aged 15–24 years. Chlamydia diagnoses also decreased among gay, bisexual and other men who have sex with men (gbMSM).

From 2023 to 2024, total chlamydia tests decreased by 1%. Among young people aged 15–24 years, tests declined by 11%, while tests increased by 3% among > 25-years-olds. In females, tests decreased by 3% overall and by 11% in those aged 15–24 years; in males, tests increased by 1% overall and decreased by 11% in 15–24-year-olds. Test positivity decreased from 5.9% to 5.2% overall and from 10.6% to 9.8% in those aged 15–24-years.

Notably, testing activity changed in recent years. Since 2020, online testing has been the most common test setting among young people, surpassing tests by specialist sexual health services and general practitioners. In 2021, a policy change shifted the focus of the National Chlamydia Screening Programme from screening all young people aged 15–24, to only females aged 15–24 years [17]. However, internal data checks do not show that this policy change is being widely implemented yet.

Internal UK health security agency (UK HSA) reviews of surveillance data quality and diagnoses by laboratory and assay found no indication of localised issues or changes in assay sensitivity. They noted that while reduced testing of young people can partly explain the reductions in diagnoses, chlamydia diagnoses decreased more than tests, and the most likely cause is therefore a combination of changes in who is accessing testing and lower prevalence due to behaviour changes during or since the COVID-19 pandemic.

#### Finland

Chlamydia notifications decreased by 17% between 2023 and 2024 (from 17,551 to 14,486 cases) (Table 1), which contrasted the steady increase in previous years (Figure 1). The decrease was observed in both males and females across age groups, mostly among those aged 20–24 years (−22%; Figure 2), and across 21 of 23 regions, ranging between 9% and 34% [12].

The number of chlamydia tests in 2024 remained comparable to 2023. A national analysis of data from six major laboratories, which use three different commercial assays to diagnose both chlamydia and gonorrhoea that did not change over the analysed period, showed decreases across the six laboratories in both the number of positive tests and positivity rate. No other changes were reported in testing policies or prevention measures in 2024.

#### Ireland

In 2024, Ireland observed a 16% decrease in chlamydia notifications compared with 2023 (from 13,699 to 11,524 cases) (Table 1), 13% and 19% among males and females, respectively [8]. This decrease was reported in all health regions. Notably, among those aged 15–24 years, cases declined by 21% in both males and females (Table 1), with the largest reduction of 28% among males aged 15–19 years (Figure 2) [8]. Preliminary data for 2025 indicate that the decrease continued into the first 6 months of 2025, with a 12% drop compared with the last 6 months of 2024.

The observed lower case numbers followed increases in chlamydia notifications in 2022 and 2023 (Figure 1), which were partially linked to the roll-out of a national STI testing service, launched in October 2022, that targets asymptomatic individuals and offers free home testing [8]. Between 2023 and 2024, the chlamydia positivity rate from the home testing service declined from 7.1% to 6.1%. This service accounted for 48% of chlamydia notifications in 2024, up from 43% in 2023 [8].

Chlamydia testing recommendations remained unchanged between 2023 and 2024 in Ireland. Since June 2024, treatment for most low-complexity chlamydia cases diagnosed through the national free home STI testing service have been managed within the service, shortening time-to-treatment compared with referrals to sexual health clinics. Authorities also reported targeted sexual health promotion campaigns for those under 25 years of age that have been carried out since late 2023.

#### The Netherlands

Chlamydia cases reported from the sentinel surveillance of sexual health clinics (SHCs) decreased by 16% in 2024 compared with 2023 (from 24,048 to 20,174 cases) (Table 1), reversing the previous increasing trend [18] (Figure 1). This decrease occurred across most age groups, with a predominant decrease in those aged 20–24 years (−21%) (Figure 2). The decrease was registered across all regions and varied by population subgroup: 23% among women, 13% among heterosexual men, 15% in MSM attending pre-exposure prophylaxis (PrEP) consultations and 6% in MSM attending regular consultations. National investigations found no association with education level or migration background.

Chlamydia testing decreased by 9% across SHCs in 2024 compared with 2023, varying from 3% to 25% declines across subgroups. This was accompanied by reduced positivity rates: from 16.8% to 15.3% among women, 19.6% to 19.2% among heterosexual men, 9.1% to 8.3% in MSM attending PrEP consultations and 10.2% to 9.3% in regular MSM consultations. Decreases in positivity rate were reported across all regions. Among women and heterosexual men, positivity rates had already decreased between 2022 and 2023 [18]. In 2024, SHCs discontinued routine pharyngeal testing for chlamydia; however, positivity rates still declined when looking only at anorectal or urogenital site tests [18].

#### Norway

Notified chlamydia cases decreased by 18% in 2024 compared with 2023 (from 28,137 to 23,100 cases) (Table 1). Decreases were seen across all counties, with notifications declining by 14% in males and 20% in females, respectively [6]. The largest reduction was among those aged 20–24 years, including 22% in females and 19% in males (Figure 2) [6]. Previously, in 2023, a decrease had been observed among all individuals aged 20–29 years, while a slight increase was seen among young people aged 15–19 years [19].

Chlamydia testing activity decreased in 2024 in Norway, mostly due to fewer tests performed among people younger than 25 years [6]. Positivity rates decreased from 9.0% to 7.6% in females and from 14.9% to 13.3% in males aged 20–24 years, respectively. Testing recommendations were not changed between 2023 and 2024, however, targeted public health campaigns launched in autumn 2022 in response to increasing gonorrhoea infections in young people may have influenced recent trends. These campaigns aimed to increase awareness, promote condom use and encourage testing, especially among students. Although condom use is generally low, the Norwegian Directorate of Health has recorded an increase in 2023 in orders for free condoms through the national scheme [20].

#### Sweden

Chlamydia cases decreased by 18% between 2023 and 2024 (from 32,298 to 26,392 cases) (Table 1). The decline occurred in both males and females across the younger age groups, with the largest reduction in those aged 20–24 years (−24%) (Figure 2). Heterosexual transmission remained predominant, and the proportion of cases among men reported as MSM was stable compared with 2023 (17%). Decreases in chlamydia incidence were observed in 19 of 21 regions, ranging from 10% to 42%. National experts noted that although rates have declined by an average of 3.4% annually in the last 10-year period, this sharp drop was unexpected. Preliminary data show that the decrease continued into the first 6 months of 2025.

In 2024, the number of chlamydia tests remained stable compared with 2023 but represented an approximate 8% increase since 2022. The highest testing volumes were in females and people aged 20–24 years. The proportion of positive tests declined from 5.4% in 2023 to 4.4% in 2024 [7]. There were no changes in testing practices, laboratory methods, public health interventions or campaigns that could explain the decrease in chlamydia. Authorities consider it likely that the decrease is driven by several interacting factors that have influenced sexual behaviour and contact patterns among young people, and the reasons for the sharp chlamydia decrease are under investigation [7].

## Observed declines relative to pre-COVID-19 pandemic and counterfactual chlamydia trends

The ECDC conducted a retrospective analysis to compare chlamydia cases reported in 2024 with those reported in the years before the COVID-19 pandemic (2015–2019) in each country. 

### Methodology

A negative binomial model was fitted to annual chlamydia case counts from 2015 to 2019 by country for the two age groups with the greatest decline (15–19 and 20–24 years). Model coefficients were reported as percentage changes in annual case counts with 95% confidence intervals (CIs) to quantify the uncertainty in the estimated trend pre-COVID-19. Counterfactuals predictions for 2020 to 2024 were then calculated for each country by extending the pre-COVID-19 trend in reported case counts. The 95% prediction intervals (PIs) were calculated to account for the uncertainty and variability in the predictions, using the R package ciTools (v0.6.1) in RStudio 2024.04.2.

### Comparative estimates

**Figure 3:**
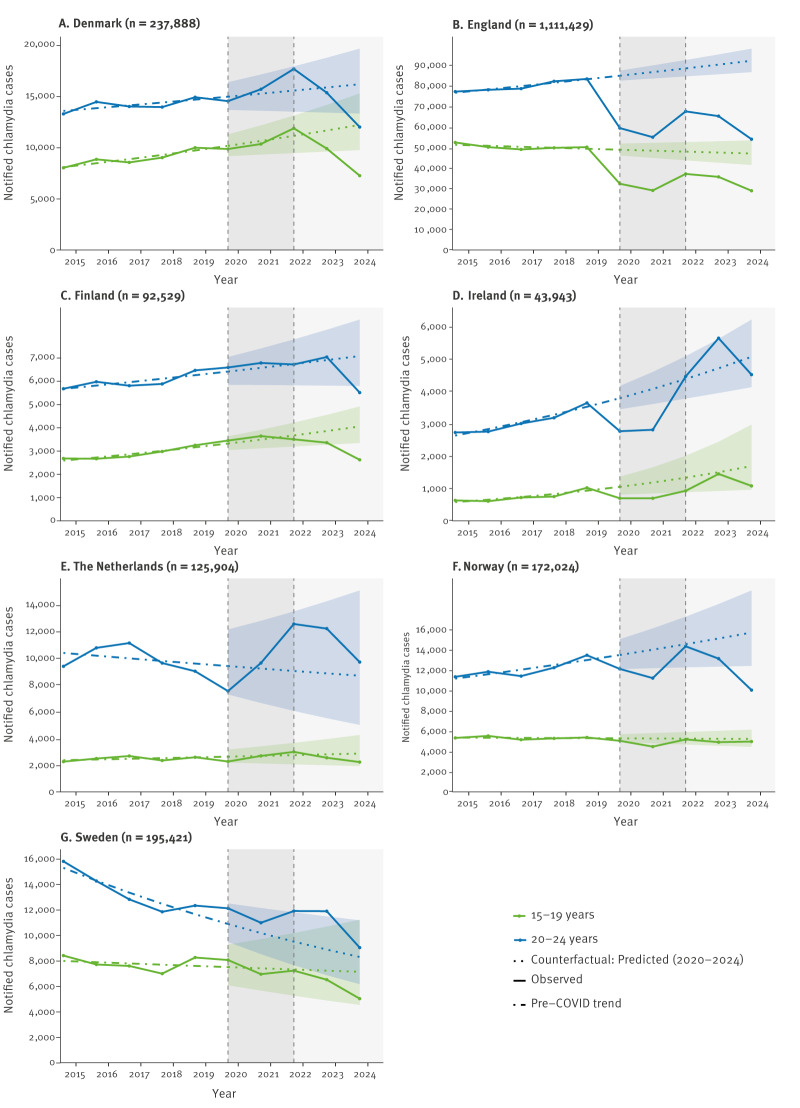
Number of chlamydia case notifications and counterfactual scenario among individuals aged 15–19 years and 20–24 years, seven countries, 2015–2024 (n = 1,979,138)

Model estimates indicated that pre-pandemic trends varied across countries and age groups (Figure 3). Specifically, among those aged 15–19 years, the model estimated that pre-pandemic chlamydia cases increased in Denmark and Finland (by 5% per year), and in Ireland (by 13% per year); in 2024, observed cases fell below the levels predicted by these increasing trends in Denmark and Finland, but remained within the counterfactual 95% PI in Ireland (Figure 3, Table 2). Meanwhile, pre-pandemic trends were stable in England, the Netherlands, Norway and Sweden; in 2024, observed cases fell below the 95% PI in England but remained within the counterfactual 95% PI in the Netherlands, Norway and Sweden.

Grey area: COVID-19 pandemic years 2020–2022; Shaded areas in blue and green: 95% prediction intervals

Estimated pre‑pandemic trends (2015–2019), expressed as relative change in annual case counts can be seen in Table 2.

**Table 2:** Pre-pandemic annual change in chlamydia case counts (2015–2019) and comparison of observed cases in 2024 with counterfactual predictions, by country and age group, seven countries, 2015–2024 (n = 1,979,138)

Country	Age group (years)	Pre-pandemic annual change in reported chlamydia case counts, 2015–2019	95% CI	Observed cases in 2024 vs counterfactual (95% PI)
Denmark	15–19	1.05	1.03–1.07	**Below PI**
	20–24	1.02	1.00–1.04	**Below PI**
England	15–19	0.99	0.98–1.00	**Below PI**
	20–24	1.02	1.02–1.03	**Below PI**
Finland	15–19	1.05	1.03–1.07	**Below PI**
	20–24	1.03	1.01–1.04	**Below PI**
Ireland	15–19	1.13	1.07–1.18	Within PI
	20–24	1.08	1.06–1.10	Within PI
The Netherlands	15–19	1.02	0.99–1.06	Within PI
	20–24	0.98	0.94–1.03	Within PI
Norway	15–19	1.00	0.98–1.01	Within PI
	20–24	1.04	1.02–1.06	**Below PI**
Sweden	15–19	0.99	0.95–1.03	Within PI
	20–24	0.93	0.91–0.96	Within PI

CI: confidence interval; PI: prediction interval.

Pre-pandemic trends were estimated using negative binomial regression based on annual case counts from 2015–2019. They represent annual changes in reported case counts and should not be interpreted as incidence rate ratios, as no population offset was included. Counterfactual predictions for 2020–2024 were calculated by extending the pre-pandemic trend in reported case counts. 'Below PI' indicates that observed case numbers in 2024 fell below the lower bound of the 95% PI.

Among those aged 20–24 years, estimates suggest that chlamydia cases increased before the COVID-19 pandemic in Denmark and England (by 2% per year), in Finland (by 3% per year), in Ireland (by 8% per year) and in Norway (by 4% per year); in 2024, observed cases fell below the levels predicted by these increasing trends in Denmark, England, Finland and Norway, but remained within the expected range in Ireland (Figure 3, Table 2). Trends were constant in the Netherlands and decreased in Sweden (by 7% per year); in 2024, observed cases remained within the counterfactual 95% PI in both countries.

## Discussion

For 2024, the seven participating countries reported decreases in chlamydia case notifications. Decreases were mainly among individuals aged 15–24 years, especially among young women aged 20–24 years. In Denmark, England and Finland, the number of cases in 2024 were lower than expected based on the counterfactual prediction among individuals aged 15–24 years, indicating a marked departure from the increasing pre-pandemic trends. In Norway, this was only the case among those aged 20–24 years. The decreases continued a previous decline in cases since 2022 among those aged 15–24 years in Denmark, England, the Netherlands and Norway. Finland and Sweden saw decreases from 2022 to 2024 in chlamydia cases among individuals aged 20–24 years, and Sweden to a lesser extent also among those aged 15–19 years. In contrast, Ireland had observed increases in 2023 compared to 2022, with case numbers peaking in 2023.

Data presented here for chlamydia vary from notification trends of other bacterial STIs reported between 2023 and 2024 by the participating countries. Ireland, England, Norway and Sweden, saw concurrent gonorrhoea notification decreases, although in Norway this was only in women, as cases among MSM continued to rise, and in Sweden only among individuals aged 15–19 years and 20–24 years [4,6,8,21]. England and Ireland also noted decreases in gonorrhoea among individuals aged 15–19 years and 20–24 years [4,8]. Denmark and the Netherlands reported stable gonorrhoea notifications after a sharp increase in 2022 [18,22]. Meanwhile, Finland reported a substantial overall increase in gonorrhoea cases between 2023 and 2024 [23].

Overall, the observed chlamydia decreases in these countries were unexpected in their magnitude and generated several hypotheses.

Past declines in chlamydia notifications in Sweden (2006) and Finland (2019) were linked to two distinct diagnostic escape variants that evaded detection by certain NAATs, causing under-reporting due to false negative results until assays were updated [24-26]. On both occasions, the decreases were geographically uneven, occurring in counties using the affected laboratory tests [24,25]. In contrast, the 2024 pattern shows declines in almost all subnational regions of the affected countries. Moreover, national investigations in England and Finland documented declines in positivity rates regardless of the commercial assay used. With multiple diagnostic platforms in use, a single *C. trachomatis* variant escaping detection across all regions appears unlikely.

Another hypothesis is that of a surveillance artefact. The seven countries have well-established, robust STI surveillance systems that reliably monitor trends by sex and age group. No changes were reported in notification systems or potential under-reporting differentially impacting 2024, making a reporting artefact for the 2024 data unlikely in any of these countries.

In some countries, the decline in chlamydia notifications was accompanied by reduced testing. Denmark, England the Netherlands and Norway reported reduced overall testing volumes. England saw the largest reductions among people aged under 25 years. In young people in England, testing decreased across settings, but the largest decreases were seen in online testing services. In Norway, reductions were also concentrated among those aged under 25 years. In Ireland, total testing numbers are unknown.

While changes in testing volumes varied, all countries observed a decrease in test positivity, suggesting a possible reduction in prevalence within the tested population. In England, the lower test positivity occurred alongside a large-scale shift towards online testing services since 2020 (particularly among young people), where uptake is reportedly lower among individuals at higher risk of infection compared with face-to-face testing [27-29]. Thus, changes in the tested population, with fewer individuals at high risk of *C. trachomatis* infection being tested, could have resulted in the decreased diagnoses and test positivity in England. This may also be valid in other countries that implemented testing services with a widened reach.

The use of doxycycline post-exposure prophylaxis (doxy-PEP) could plausibly contribute to the reduction in chlamydia in some population groups. In Ireland, the publication of national interim guidelines on doxy-PEP in 2024 has probably increased doxy-PEP use among gbMSM attending sexual health clinics [30]. Its impact is, however, difficult to estimate as data on doxy-PEP uptake are not routinely collected. Nevertheless, considering that doxy-PEP use is primarily recommended for gbMSM, it is unlikely to have influenced heterosexual transmission of chlamydia in Ireland.

Chlamydia decreases in the seven countries could also reflect the impact of broad interventions targeting young people, providing opportunities and incentives, or enhancing proficiencies that promote sexual health. The seven countries reported unprecedented increases in gonorrhoea notifications in 2022 and 2023 that primarily affected young women (20–24 years) and young heterosexual men in the six EU/EEA countries [31], and women aged 15–24 years in England [4,32]. In response, many countries implemented targeted interventions for young people, including awareness campaigns, condom promotion, expanded STI testing (including online), outreach via social media, reinforcement of adherence to clinical guidelines, and strengthened partner notification services. In countries that carried out these interventions, gonorrhoea prevention actions among young people could have had a collateral effect on chlamydia notifications, although the specific mechanisms remain uncertain as intervention components varied between countries. Concurrent decreases in gonorrhoea cases among young people were observed in 2024 in England, Ireland, Norway and Sweden.

Alternatively, recent decreases in chlamydia among young people could also reflect behavioural changes, possibly arising from evolving social norms or a generational shift towards improved sexual health promotion. A 2024 online survey across Nordic countries reported mixed trends of condom use among young people, a key behavioural indicator [33]. In the Netherlands, a 2023 survey showed a decline in condom use at sexual debut among respondents aged 13–25 years [34] and in Ireland, a 2022 survey found that condom use at last intercourse had dropped [35]. Contrasting these declines, the Netherlands reported increased sexual health literacy [34]. Other surveys noted reductions in adolescent risk behaviours, including later sexual debut among adolescents in Europe and lower alcohol consumption in high-income countries [36,37]. Insights into sexual behaviour in the UK are expected in the upcoming National Survey of Sexual Attitudes and Lifestyles-4 (Natsal-4). The contribution of changes in sexual behaviour and generational norms to recent STI trends remains unclear and merits further investigation, particularly given the heterogeneity of trends between STIs.

The observed trends in chlamydia among young people could also be due to a long-term impact of the COVID-19 pandemic restrictions on population prevalence. Restrictions such as school closure and social distancing could have disrupted sexual networks, reducing transmission and chlamydia prevalence. Several studies reported changes in sexual behaviours among certain populations during the pandemic [38-40]. Findings from the National Survey of Sexual Attitudes and Lifestyle (Natsal)-COVID survey of 18–59-year-olds in Great Britain found reduced sexual risk behaviour in young people [40]. Delayed sexual debut has also been suggested in some young people [38]. However, as these surveys did not include those younger than 18 years, they may not fully represent behaviours of the current cohort of 15–24-year-olds. After restrictions lifted between 2021 and 2022, a return to social mixing and more regular testing would have initially increased infection rates, then decreased and potentially stabilised at lower levels. Evidence for this hypothesis in the population of interest is currently lacking. However, a modelling study among MSM aged 15–65 years in the United States projected that 18 months of COVID-19-related sexual distancing and service disruption would reduce gonorrhoea and chlamydia incidence, followed by a brief rebound and continued decline for over 5 years [41].

The analyses presented here had some limitations. The recent decrease in chlamydia cases is based on preliminary 2024 data that seven countries present here in a format of a descriptive comparison among countries. Only aggregate counts and percentage changes were available, preventing formal significance testing. Hypotheses presented should be formally investigated with suitable methods.

Caution is advised when comparing trends across countries because surveillance system configurations differ and data completeness varies. The chlamydia decreases observed in 2024 represent only a single-year percentage change and any inference about longer-term trends would require continued follow-up. Importantly, changes in notified cases alone could reflect changes in testing rather than incidence. The robustness of the model predictions could be enhanced by increasing the number of data points, for example, by using monthly data. In addition, the counterfactual prediction assumed stable conditions, which we know were disrupted during the COVID-19 pandemic, and some countries changed testing services. Furthermore, several countries (Denmark, Finland, Ireland and Norway) do not collect data on mode of transmission. It was thus not possible to assess the contribution of changes in chlamydia diagnoses among transmission groups, such as heterosexual transmission and among MSM. Up-to-date comparable behavioural data were insufficient to put the results into context regarding changing sexual practises or networks.

ECDC in collaboration with the European STI Surveillance Network is developing EU/EEA-level chlamydia surveillance standards to improve data quality and cross-country comparability.

## Conclusion

Six EU/EEA countries and England reported declines in chlamydia cases in 2024, predominantly among young people. No major surveillance changes were identified and changes in testing policies targeting young people do not fully explain the observed declines. Possible drivers include a combination of sexual health promotion campaigns, changes in the risk profile of those accessing testing and long-term impacts of COVID-19. While variations across national contexts may favour country-specific hypotheses, the similar timing and pattern of the changes suggests a possible common driver. If the observed decreases reflected a true reduction in chlamydia infection among young populations in the seven countries, it remains uncertain whether these declines will be sustained over time. Ongoing surveillance, reliable and updated behavioural data, and improved understanding of sexual networks are needed to support more accurate interpretation.

## Data Availability

Data can be accessed on request through: Access to EU/EEA surveillance data for third parties.

## Statement

The UK is not a member of ECDC and its involvement in this publication stems from the Trade and Cooperation Agreement and ECDC-UKHSA Memorandum of Understanding.
